# Letter from the Editor in Chief

**DOI:** 10.19102/icrm.2026.17041

**Published:** 2026-04-15

**Authors:** Devi Nair


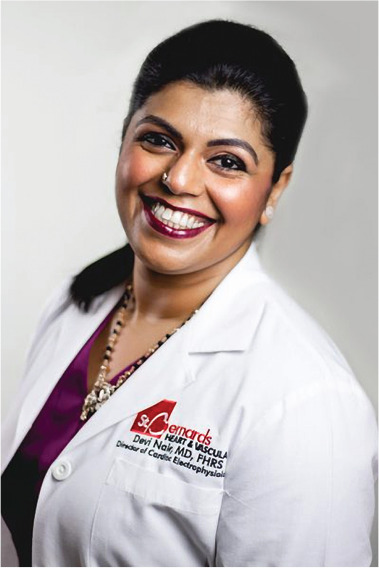


Dear Colleagues,

Welcome to the April 2026 issue of *The Journal of Innovations in Cardiac Rhythm Management*.

April was a significant month for our community. Our field gathered April 23^rd^–26^th^ at McCormick Place in Chicago for Heart Rhythm 2026, the 47th annual scientific sessions of the Heart Rhythm Society, where more than 10,000 colleagues from across the globe came together to share the latest in basic, translational, and clinical cardiac electrophysiology. The program once again reflected the breadth of our discipline, the continued maturation of pulsed field ablation, refinements in conduction system pacing, the role of artificial intelligence (AI) in mapping and signal analysis, the management of complex ventricular substrates in mechanically supported patients, and the expanding interface between devices and heart failure care. On Saturday evening, the second annual Heart Rhythm Gala brought our community together in support of advocacy, research, and education through Heart Rhythm Advocates, a fitting reminder that scientific progress is sustained by collective commitment that extends well beyond the laboratory and the electrophysiology suite.

The manuscripts in this issue mirror that breadth, spanning device innovation, advanced mapping in mechanically supported circulation, AI-guided substrate ablation, mechanistic tracings, and the emerging voices of our trainees.

Pande et al.^1^ report what they believe to be the first documented case of left bundle branch area pacing achieved with a single-coil defibrillator lead (Reliance 4-Front; Boston Scientific, Marlborough, MA, USA) positioned using a manually shaped stylet in a 52-year-old man with severe nonischemic cardiomyopathy and recurrent ventricular tachycardia. By unifying physiologic pacing and defibrillation in a single lead, the authors highlight both the promise of conduction system pacing in this population and the technical creativity required in environments where dedicated delivery sheaths are not yet available. The case is also a candid reminder that left bundle branch area pacing with a defibrillator lead remains an investigational frontier, awaiting purpose-built tools and chronic outcome data before broader adoption.

O’Leary et al.^2^ present two challenging cases of HeartMate 3™–associated (Abbott, Chicago, IL, USA) ventricular tachycardia successfully treated with substrate-based ablation using the TriDeca™ high-density linear mapping catheter (Stereotaxis, St. Louis, MO, USA). Their work elegantly demonstrates how closely spaced 1-mm electrodes, with their high-fidelity near-field recordings, can delineate slow conduction channels even in the presence of electromagnetic interference and the distorted ventricular geometry imposed by continuous-flow mechanical support. Equally important are the mechanical advantages of a linear catheter that can be gently curled within the ventricle to avoid suction near the inflow cannula, safety considerations that are inseparable from electrical performance in this anatomically uncompromising substrate.

In an original research contribution, my team^3^ describes a pilot, prospective, single-center experience with AI-guided extra-pulmonary vein repeat ablation using the pentaspline pulsed field catheter. Twenty-five patients with recurrent persistent atrial fibrillation underwent personalized ablation of spatiotemporal dispersion identified by the Volta AF-Xplorer™ algorithm (Volta Medical, Providence, RI, USA). The mean procedure time was approximately 75 min, performed entirely without fluoroscopy; sinus rhythm conversion was achieved in 92% of patients, and 88% remained free of any atrial arrhythmia at 6 months. While preliminary, the findings suggest that the procedural efficiency of a large-footprint pulsed field ablation catheter can be married to a substrate-tailored workflow without prohibitive efficiency cost, an open question since TAILORED-AF (“Tailored vs. Anatomical Ablation Strategy for Persistent Atrial Fibrillation”) demonstrated the value of personalized strategies but at considerable procedural times using point-by-point radiofrequency.

In this month’s Tracing of the Month, Korkmaz et al.^4^ dissect a wide QRS complex tachycardia in a young woman with prior accessory pathway and atrioventricular nodal re-entrant tachycardia ablations. Through meticulous analysis of differential responses to early and late premature atrial contractions delivered from the coronary sinus, the authors arrive at a diagnosis of antidromic nodofascicular/nodoventricular re-entrant tachycardia. The case is a masterclass in interval-based reasoning and a reminder that, even in the era of high-density mapping and panoramic imaging, the proximal coronary sinus recording and the timing of a well-placed premature atrial contraction remain among our most powerful diagnostic instruments.

This issue also features the conference proceedings from the 2025 Electrophysiology Fellows Summit & Arrhythmia Scholars Program held November 7–9, 2025, in Reston, VA, introduced in a letter^5^ from program directors Drs. William Sauer and Wendy Tzou. The three case competition finalists, Drs. Hasan Munshi (St. Joseph’s University Medical Center), Jonathan Gordon (Rush University Medical Center), and Sittinun Thangjui (West Virginia University), presented^6–8^ cases that span the contemporary landscape of complex ablation: persistent right phrenic nerve palsy following pulsed field ablation; Purkinje fiber catheter ablation for incessant ventricular fibrillation in a left ventricular assist device (LVAD) recipient; and the winning case, a first reported combined pulsed field and radiofrequency ventricular tachycardia ablation using the Sphere-9™ catheter (Medtronic, Minneapolis, MN, USA) guided by EnSite™ (Abbott) impedance–based mapping in a durable LVAD patient. Taken together, these reports remind us that the next generation of electrophysiologists is already engaging the field’s hardest problems with curiosity, rigor, and an instinct for innovation.

This April 2026 issue traces an arc from sophisticated case-based reasoning to procedural innovation in some of the most challenging substrates we encounter and from established mechanisms to emerging energy modalities. The very questions explored in these pages—lesion durability, physiologic pacing in complex anatomy, mechanical circulatory support and arrhythmogenesis, AI-augmented decision-making, and the careful interpretation of intracardiac signals—were precisely the ones that animated the podia and the corridor conversations at McCormick Place.

I am grateful, as always, to our authors, reviewers, and editorial team for their work, and to you, our readers, for your continued engagement with the journal.

Warm regards,


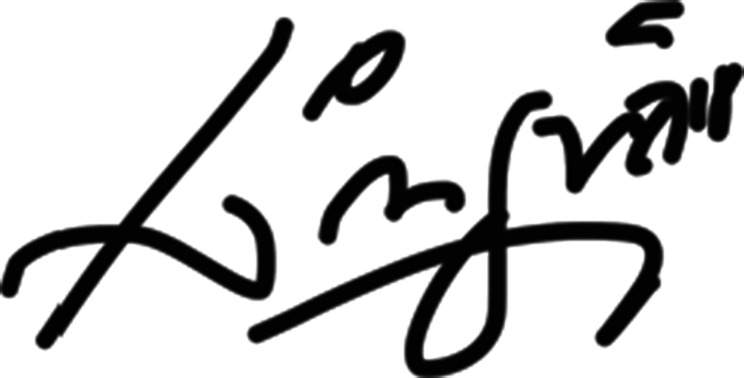


Dr. Devi Nair, md, facc, fhrs

Editor-in-Chief

*The Journal of Innovations in Cardiac Rhythm Management*

Director of the Cardiac Electrophysiology & Research,

St. Bernard’s Heart & Vascular Center, Jonesboro, AR, USA

White River Medical Center, Batesville, AR, USA

President/CEO, Arrhythmia Research Group

Clinical Adjunct Professor, University of Arkansas for Medical Sciences

Governor, Arkansas Chapter of the American College of Cardiology

drdgnair@gmail.com
